# Antifungal potential of naphthoquinone derivatives: screening of shikonin-based compounds and mechanistic insights into 5,8-dihydroxy-1,4-naphthoquinone against *Candida albicans in vitro* and *in vivo*

**DOI:** 10.1128/spectrum.02438-25

**Published:** 2026-02-27

**Authors:** Qingqing Chen, Minkai Yang, Xiaohui Lai, Jiabao Hu, Aliya Fazal, Yahan Zhang, Xiaoran Lv, Jiaxuan Xiao, Zheng Fan, Zichen Pan, Tongming Yin, Shucun Sun, Guihua Lu, Jinliang Qi, Hongyan Lin, Zhongling Wen, Yonghua Yang, Hongwei Han

**Affiliations:** 1 State Key Laboratory of Pharmaceutical Biotechnology, Institute of Plant Molecular Biology, School of Life Sciences, Nanjing University 12581, Nanjing, Jiangsu, China; 2 Co-Innovation Center for Sustainable Forestry in Southern China, Nanjing Forestry University 74584, Nanjing, Jiangsu, China; 3 School of Pharmacy, Changzhou University 12412, Changzhou, Jiangsu, China; Debreceni Egyetem, Debrecen, Hungary

**Keywords:** naphthoquinone derivatives, antifungal, *Candida albicans*, biofilm disruption, glycolysis inhibition, oxidative stress

## Abstract

Invasive fungal infections (IFIs), predominantly caused by *Candida albicans*, are a significant threat to immunocompromised individuals. The emergence of drug-resistant strains has intensified the need for novel antifungal agents. Natural naphthoquinones, including 5,8-dihydroxy-1,4-naphthoquinone (DHNQ, also as PNP-02), have broad-spectrum antimicrobial properties, but their antifungal potential against *C. albicans* remains underexplored. This study evaluates the antifungal activity of DHNQ derivatives and elucidates their mechanisms of action. The antifungal properties of these compounds were evaluated using the Kirby-Bauer disk diffusion method, broth microdilution assays, and phenotypic screening. DHNQ was identified as the most effective compound, and further investigation focused on its effects on *C. albicans* growth, biofilm formation, hyphal development, and underlying mechanisms, including oxidative stress induction and mitochondrial dysfunction. In a murine candidiasis model, DHNQ significantly reduced the fungal burden in both the kidneys and the skin, with a minimum inhibitory concentration (MIC) ranging from 2 to 8 μg/mL, exceeding the activity of fluconazole against clinical isolates of fluconazole-resistant *C. albicans* strains by over 32 times. Mechanistic investigations revealed that DHNQ exerts its antifungal effects through a multi-pronged approach: inhibiting glycolysis, disrupting biofilm and hyphal formation, and inducing oxidative stress-mediated mitochondrial dysfunction. Notably, DHNQ exhibited low cytotoxicity *in vitro* and no observable toxicity *in vivo*. These properties make it a promising lead molecule for future optimization and development of treatments for *C. albicans* infections, pending crucial evaluations of its selectivity and safety in host environments.

**IMPORTANCE:**

In summary, this study demonstrated that DHNQ exhibits potent and broad-spectrum antifungal activity, showing significant efficacy against *C. albicans* both *in vitro* and *in vivo*. Unlike conventional antifungals, DHNQ disrupted the virulence of *C. albicans* by inhibiting glycolysis, suppressing biofilm formation, and inducing oxidative stress-mediated mitochondrial dysfunction. These findings not only highlight the promising potential of DHNQ as a treatment for *C. albicans* infections but also provide critical insights that may facilitate the development of new antifungal agents.

## INTRODUCTION

*Candida* species are the major cause of IFIs in severely immunocompromised populations (1). The biofilm form of *C. albicans*, which exhibits strong drug resistance and adheres to medical devices or host tissues, significantly complicates clinical cures (2, 3). The emergence and spread of resistant *C. albicans* strains in recent years have highlighted an urgent need to develop effective therapeutics (4, 5). The understanding of the epidemiology, virulence factors, and drug resistance of *C. albicans* is limited (6, 7). The discovery of active compounds through screening of clinical antifungal candidates or recognized antifungal drugs has been challenging (6, 8). Drug repurposing of marketed drugs has identified lead compounds or synergistic drug combinations, but their limited potency and selectivity have hindered direct application (9, 10).

Plant natural products play a vital role in the development of new pharmaceuticals. The successful development and application of compounds such as artemisinin and its derivatives, quinine, and oridonin—the principal component of the antiviral drug Tamiflu—have established these natural products and their derivatives as crucial scaffolds for the creation of new anti-infective drugs (11). Ancient medical texts in China document the use of *Lithospermum* for treating conditions, such as carbuncles and rashes, and its therapeutic efficacy has now been validated. For example, a cream derived from *Lithospermum* root extract, known as Shiunko, is utilized in Japan to treat burns and hemorrhoids (12). Additionally, a foot fungus ointment containing deoxylithospermine as the active ingredient received a patent in the 1980s, further confirming the antifungal activity of shikonin in practical applications (13). Research revealed that shikonin and its derivatives exhibited significant antibacterial activity against various pathogenic strains (14–16). Shikonin has shown excellent effects in inhibiting *C. albicans* biofilms and related gene expression and has significantly reduced fungal burden in mice with vulvovaginal candidiasis *in vivo* experiments (14, 17). Despite these promising findings, research on the antifungal properties of shikonin compounds remains limited. There is a notable lack of broad-spectrum antifungal activity screening, as well as in-depth and systematic studies on antifungal mechanisms.

In this study, we aim to identify and assess the antifungal properties of nine natural naphthoquinones from Zicao roots and their core structure-5,8-dihydroxy-1,4-naphthoquinone (DHNQ, PNP-02) against five common pathogenic fungi: *C. albicans*, *C. neoformans*, *A. fumigatus*, *A. niger*, and *T. rubrum*. Through proteomic analysis and *in vivo* infection studies using mice infected with *C. albicans*, we seek to elucidate the mechanisms of action of these compounds and provide critical insights that may facilitate the development of new therapeutic agents and improve clinical strategies for treating fungal infections.

## MATERIALS AND METHODS

### Materials

**Fig 1:**
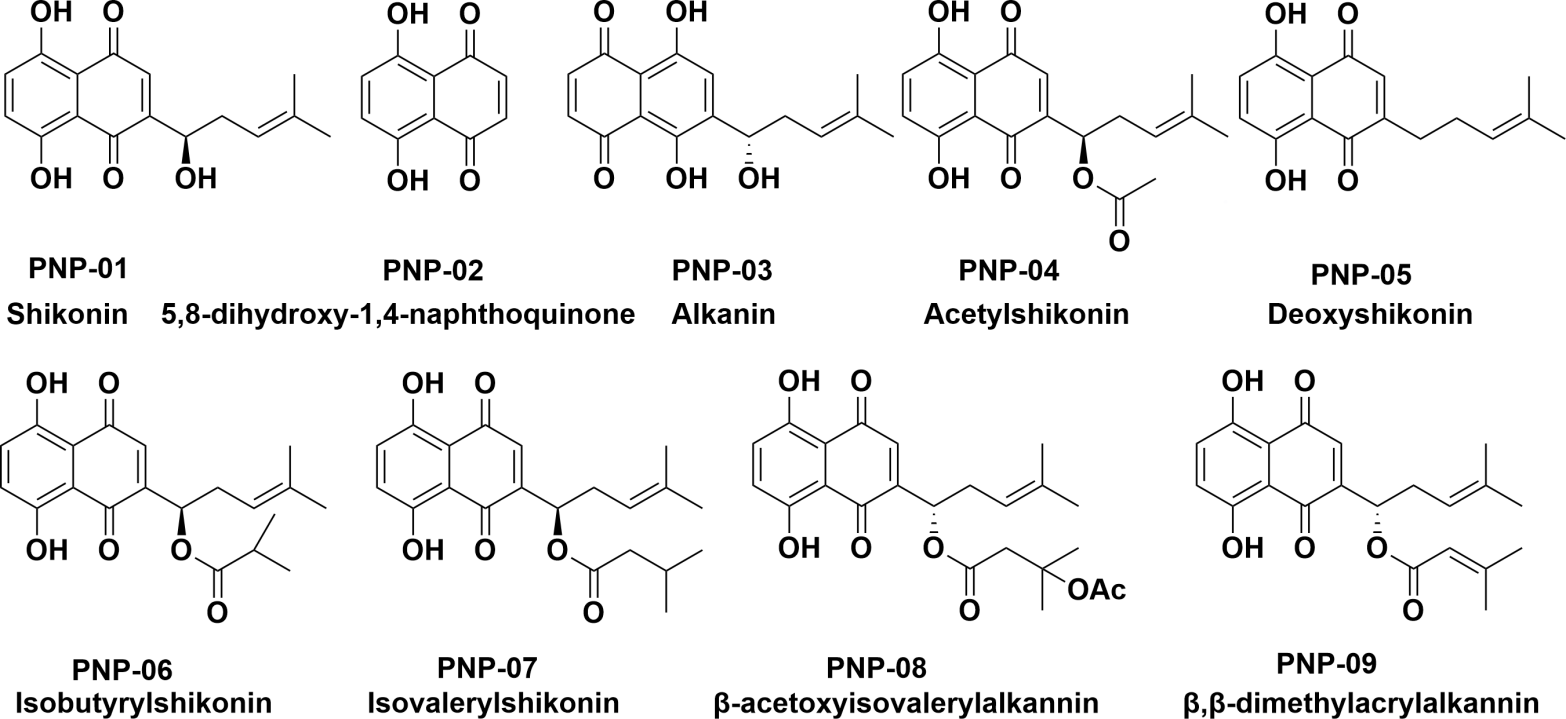
Chemical structure of the nine natural shikonin compounds.

*C. albicans* ATCC10231, *C. neoformans* H99, and *A. fumigatus* KU80 were kindly donated by Dr. Yuanwei Zhang from Nanjing Normal University. *A. niger* is from the State Key Laboratory of Pharmaceutical Biotechnology, Nanjing University. *T. rubrum* BNCC340195 was purchased from Henan Province Industrial Microbial Strain Engineering Technology Research Center. Eleven clinical isolates of *C. albican*s (C1-13, C1-14, C1-17, C1-20, C1-21, C1-22, C1-23, C1-24, C1-27, 06-37, and 09-07) were purchased from the Hospital of Dermatology, Chinese Academy of Medical Sciences. The natural shikonin compounds (purity ≥ 98%) used in this experiment (Fig. 1) were all pre-synthesized or isolated non-commercial small molecules, dissolved in DMSO for *in vitro* experiments and prepared into a 0.5% solution of CMC-Na/saline water for *in vivo* experiments. Fluconazole (FCZ) was obtained from Yuanye Biotechnology Co., Ltd. (Shanghai, China). Amphotericin B (AMB) and itraconazole (ITC) were obtained from Maikelin Biochemical Technology Co., Ltd. (Shanghai, China). Terbinafine (TBF) was obtained from Shanghai Jizhi Biochemical Technology Co., Ltd.

### Antifungal susceptibility testing

Primary antifungal activity screening of nine natural shikonin compounds was conducted using the Kirby-Bauer paper diffusion method, as judged by the inhibitory ring diameter.

The minimal inhibitory concentrations (MICs) of shikonin compounds for *Aspergillus* and dermatophyte were determined according to the M38 Ed3 (18) protocol of the Clinical and Laboratory Standards Institute (CLSI), and for yeasts using M27 Ed4 guidelines (18, 19). Standard positive controls were established using commercial antifungal drugs: AMB, ITC, and TBF served as positive controls for yeasts (including *C. albicans* and *C. neoformans*), *Aspergillus* species (comprising *A. fumigatus* and *A. niger*), and dermatophytes (e.g., *T. rubrum*), respectively. Furthermore, both FCZ and AMB also served as positive controls against clinical strains of *C. albicans*. In brief, the initial concentration of the fungal suspension in RPMI 1640 medium was 1.5–3 × 10^3^ CFU/mL (8, 20, 21). The final drug concentrations were 0.125–64 µg/mL for natural shikonin compounds; 0.025–12.8 µg/mL for AMB, ITC, and TBF; and 0.5–256 µg/mL for FCZ. Incubation conditions were tailored to each fungal species: *C. albicans* and *C. neoformans* were incubated in SDA medium at 30°C for 24 h; *A. niger* and *T. rubrum* were incubated in PDA medium at 27°C for 48 h and 7 days, respectively; and *A. fumigatus* was incubated in YAG medium at 37°C for 24 h. RPMI medium wells and fungal wells were used as the blank control and the positive control, respectively. The MIC was defined as the lowest concentration of an antifungal agent resulting in a 100% reduction in visible growth compared to the control (22, 23).

To determine the minimum fungicidal concentration (MFC) (the lowest concentration of compounds to completely eradicate fungi) values (23), 30 μL of fungal suspension of each well from the above MIC assay was spread on agar plate, and then, the plate was incubated at 30°C to obtain the MFC. The experiments were repeated three times independently.

### *In silico* calculations

OSIRIS DataWarrior, Molinspiration (https://www.molinspiration.com/), and AdmetSAR (http://lmmd.ecust.edu.cn) were employed to predict the molecular properties, toxicity risks, bioactivities, and pharmacokinetic characteristics of several natural shikonin compounds with relatively better antifungal activity, e.g., PNP-01, DHNQ, PNP-03, and PNP-04, while the positive drug FCZ, AMB, ITC, and TBF were also predicted.

### Cell viability

Cell viability was evaluated using the CCK-8 assay (24). Cells were seeded in 96-well plates. Different concentrations of compounds were introduced into the wells, and the plates were placed in an incubator set at 37°C. Cell viability was then assessed using the CCK-8 assay kit (Yeasen Biotechnology Co. Ltd., Shanghai, China) at 450 nm.

### Fungicidal kinetic studies

The concentration of *C. albicans* suspension was adjusted to 10^6^ CFU/mL, and different concentrations of DHNQ were added to make the final concentration of 0.5, 1, 2, and 4 μg/mL, respectively. OD_600_ of the suspension over time was continuously monitored over 12 h.

Time-kill assays were also performed. DHNQ at final concentrations of 0.5, 1, 2, 4, and 8 μg/mL was added to a concentration of 10^6^ CFU/mL of *C. albicans* suspension. Portions of cell suspensions were withdrawn at predetermined time points (2, 4, 6, 8, 12, and 24 h after incubation). The CFUs of samples in different DHNQ concentrations and time points were counted.

### Scanning electron microscopy (SEM) observation

*C. albicans* was treated with 4 μg/mL DHNQ for 4 h. Cells were collected by centrifugation (4,000 rpm for 5 min), followed by fixation (2.5% paraformaldehyde and 2% glutaraldehyde), rinsing, dehydration (30%, 50%, 70%, 80%, and 90% alcohol gradients), and drying. Then, the samples were observed by SEM.

### Propidium iodide (PI) staining analysis

The concentration of *C. albicans* suspension was adjusted to 10^6^ CFU/mL, and the cells were treated with DHNQ at various concentrations (0.5, 1, and 2 μg/mL) for 4 h with agitation (150 rpm). Then, the cells were harvested, resuspended in PBS, and stained with 10 μg/mL PI. Cells were observed using a 514 nm laser of a confocal laser scanning microscope (Zeiss).

### Detection of soluble protein leakage

After treatment with DHNQ at various concentrations (0.5, 1, and 2 μg/mL) for 4 h, *C. albicans* cells were centrifuged (8,000 × *g* for 10 min), and the supernatant was collected to determine the soluble protein content released by the ruptured *C. albicans* cells using the BCA Protein Quantification Kit (Yeasen Biotechnology, Shanghai), following the manufacturer’s instructions.

### Detection of chitin and β-3-glucan in cell wall

*C. albicans* was treated with DHNQ at final concentrations of 1, 2, 4, and 8 μg/mL for 4 h at 30°C. After cells were collected by centrifugation (4,000 rpm for 5 min) and washed with PBS, 15 μL calcofluor white (CFW) was added to dye chitin, and the cells were incubated in the dark at room temperature for 30 min. As for β-3-glucan, aniline blue at a final concentration of 0.1% was added to the cells, followed by incubation in the dark at 80°C for 15 min and cooling to room temperature. Then, the cells were observed by confocal laser scanning microscope, and the fluorescence intensity was detected using a fluorescence microplate (Ex = 355 nm, Em = 440 nm for the detection of CFW and Ex = 398 nm, Em = 508 nm for the detection of aniline blue).

### Sorbitol protection test

Sorbitol is an osmotic pressure stabilizer that stabilizes fungal protoplasts and reduces the fungicidal effect of cell wall damage, so the MIC of cell wall-targeting drugs could be increased in media containing sorbitol. RPMI 1640 dissolved sorbitol to a final concentration of 0.8 M. The MIC of DHNQ in the presence of sorbitol was measured and was compared to the positive drug caspofungin, which could destroy fungal cell walls by inhibiting β-1,3-glucan synthetase.

### Proteomics analysis

The concentration of *C. albicans* ATCC1023 suspension was adjusted to 10^6^ CFU/mL and divided into a control group (untreated) and a treatment group (4 μg/mL DHNQ, incubated at 30°C for 4 h), with three biological replicates per group. After collecting the cell pellets, total proteins were extracted using RIPA lysis buffer. Following BCA quantification, 10 μg of protein was separated by SDS-PAGE and digested with trypsin to obtain peptides. LC-MS/MS analysis was performed using a Thermo Q Exactive HF mass spectrometer with the following parameters: scan range 350–1500 m/z; primary mass spectrometry resolution 45,000; secondary mass spectrometry resolution 15,000; collision energy 32 eV; and dynamic exclusion time 15 s. Differentially expressed proteins were screened using a 1.5-fold change for GO and KEGG analyses.

### Glucose consumption and intracellular ATP concentration assay

*C. albicans* was treated with DHNQ at final concentrations of 1, 2, 4, and 8 μg/mL for 4 h at 30°C. The supernatant obtained after centrifugation (4,000 rpm for 5 min) was collected and assayed for glucose consumption using a glucose colorimetric assay kit (Elabscience). Glucose consumption was measured by detecting the fluorescence of the product from the Trinder reaction, which involves hydrogen peroxide produced during the oxidation of glucose. The glucose content was calculated by detecting OD_505_ and normalizing with the protein concentration of each sample.

The intracellular ATP concentration was measured using an enhanced ATP assay kit (Beyotime) according to the manufacturer’s instructions. Cells were lysed by ultrasonication, and ATP was extracted from cells and mixed with an ATP assay working solution containing luciferase. RLU was measured by a luminometer (Tecan).

### Biofilm disruption assay

*C. albicans* cells (10^6^ CFU/mL) were added to a 96-well tissue culture plate for 1.5 h of adhesion at 37°C to form the primary biofilm. Following adhesion, the medium was aspirated so that suspension cells were removed and washed with PBS, and then, fresh medium with various concentrations of DHNQ (2, 4, 8, and 16 μg/mL) was added to the adherent cells. The plate was further incubated at 37°C for 24 h. The XTT (2,3-bis(2-methoxy-4-nitro-5-sulfophenyl)-2H-tetrazolium-5-carboxanilide) reduction assay was performed to examine the metabolic activity of the biofilms (8). To test the effect of DHNQ on *C. albicans* mature biofilm, cells were incubated at 37°C for 24 h instead of 1.5 h. Then, the biofilm was washed, and DHNQ was added as described above. The plates were incubated at 37°C for 24 h to detect the anti-mature biofilm effect of DHNQ. The disruption of biofilm was also observed by microscope after crystal violet staining (Keygen biotech).

### Hyphae formation assay

The concentration of *C. albicans* suspension was adjusted to 10^7^ CFU/mL using hyphae induction medium containing 10% FBS. Different concentrations of DHNQ were added to make final concentrations of 2, 4, 8, and 16 μg/mL, respectively. Afterward, cells were incubated at 37°C for 4 h. Hyphae were then observed using the microscope, and the hyphae length was measured using Image J.

### Biofilm cell hydrophobicity assay

The concentration of fungal suspension was adjusted to 10^7^ CFU/mL, and 600 μL of bacterial suspension was added to each well of a 24-well plate. The fungal suspension was left in an incubator at 37°C for 24 h. Then, the medium was absorbed and washed with PBS. The biofilm cells on the bottom of the two plates were gently scraped off with a cell scraper and then added to SDB medium to make biofilm cell suspension. To adjust the fungal biofilm cell suspension to an OD_600_ of 1.0, transfer 1.2 mL to a 5 mL centrifuge tube, add 300 μL of octane, and vortex violently for 3 min. Let the mixture stand at room temperature for 15 min to allow two-phase separation, then observe the upper organic phase for transparency, and take a photograph.

### Intracellular ROS measurement

The effect of DHNQ on intracellular ROS levels in *C. albicans* was examined by the fluorescent dye DCFH-DA (Meilunbio). *C. albicans* cell suspension was adjusted to 10^6^ CFU/mL and incubated with different concentrations of DHNQ (1, 2, 4, and 8 μg/mL) at 30°C for 4 h. Rosup (reactive oxygen species upregulator) is a chemically defined oxidative stress inducer commonly used as a positive control in ROS assays. Cells were collected by centrifugation (4,000 rpm for 5 min), washed with PBS, resuspended in 40 μmol/L DCFH-DA working solution, and incubated at 37°C for 25–30 min, during which time the mixture of cells and probes was inverted up and down every 3–5 min. The cells were washed with PBS three times, and the probes that did not enter the cells were fully washed to reduce the background, while the cell precipitates loaded with probes were re-suspended with 200 μL PBS. The fluorescent cells were observed using a 488 nm laser on a confocal laser scanning microscope and counted using Image J.

### Mitochondrial membrane potential measurement

The change of MMP in *C. albicans* after treatment with DHNQ was analyzed using the JC-1 fluorescent probe (Yeasen), according to the manufacturer’s instructions. *C. albicans* cell suspension was adjusted to 10^6^ CFU/mL and incubated with different concentrations of DHNQ (1, 2, 4, and 8 μg/mL) at 30°C for 4 h. Cells were collected by centrifugation (4,000 rpm for 5 min), washed with PBS, and resuspended in 500 μL PBS and 500 μL JC-1 staining working solution, followed by incubation at 37°C for 20 min. After incubation, the precipitate was collected by centrifugation (4,000 rpm for 5 min), washed twice, and resuspended in JC-1 staining buffer. Cells were observed by confocal laser scanning microscope and analyzed with a fluorescence microplate reader (Ex = 525 nm, Em = 590 nm for the detection of JC-1 aggregate and Ex = 490 nm, Em = 530 nm for the detection of JC-1 monomer). The ratio of the fluorescent intensity of JC-1 aggregate/monomer represented the mitochondrial membrane potential.

### Effects of antioxidants on the antifungal effect of DHNQ

To investigate the effect of strong antioxidants N-acetylcysteamine (NAC) and glutathione (GSH) on the antifungal activity of SK, freshly prepared NAC and GSH solutions were added to the RPMI 1640 medium to final concentrations of 0.25, 0.5, and 1 mmol/L. As described above, the NAC-containing and GSH-containing media were used in the MIC measurement for DHNQ.

### *In vivo* antifungal potency

Female 8-week-old ICR mice (weighing 18–20 g) were obtained from the Experimental Animal Center of the Jiangsu Huachuang Sino Pharm Tech. All animal experiments were approved by the Committee on Ethics of IACUC of Jiangsu Huachuang Sino Pharm Tech (Certificate SCXK-2020-0041).

### C. albicans superficial infection

All mice were anesthetized with 2% isoflurane solution, and a 1-cm^2^ skin-deep incision was made on the back of each mouse (23, 25). Then, they were randomly divided into three groups: a model group, a 15 mg/kg DHNQ treatment group, and a 30 mg/kg DHNQ treatment group. Six mice were in each group. The skin incision of mice was infected with 100 μL *C. albicans* isolate solution (i.e., 10^6^ CFU/mL). After 5 h, PBS and DHNQ (15 and 30 mg/kg) were applied, respectively. After 5 days of treatment, skin tissues were collected and homogenized with PBS, diluted, and coated in LDA plates to determine the fungal load of each infection site in each mouse. After culturing at 37°C for 48 h, live colonies were photographed with a digital camera, and the CFUs were counted.

### *C. albicans* invasive infection

Fungal cells in the exponential growth phase were collected, washed three times with PBS, and then physiological saline was used to dilute the fungal suspension to 3.0 × 10^5^ CFU/mL. ICR female mice were injected with fungal suspension (*C. albicans*, 0.2 mL) via the tail vein to create a mouse model of invasive fungal infection. Mice were dosed after 2 h. Mice in different compound groups were treated with oral medicine once a day at fixed time and remained dosed for 5 days. At the end of administration, the mice were euthanized on the 6th day, the kidneys of mice were aseptically enucleated for homogenization, and the appropriate concentrations of the homogenates were inoculated onto SDA plates and incubated for 48 h. Finally, the number of colonies generated on the SDA plate was counted, and the amounts of fungal colonies in the kidney tissue were calculated. For the killing assay, mice (six mice per group) were treated orally with DHNQ at doses of 15 mg/kg or 30 mg/kg and then monitored daily for 7 days post-infection. Survival curves were analyzed using the Kaplan–Meier method and analyzed with the log-rank test in GraphPad Prism. A *P* value< 0.01 (**) was considered statistically significant (8).

### Data statistics analysis

The results are expressed as the mean ± standard deviation (SD). All statistical analyses were performed using the unpaired Student’s *t*-test and one-way analysis of variance (ANOVA) with the GraphPad Prism 8.0 software. *P* values less than 0.05 were considered statistically significant: * *P* < 0.05, **P < 0.01, ***P < 0.001*.*

## RESULTS

### Discovery of an anti-*C. albicans* compound by phenotypic screen

**Fig 2:**
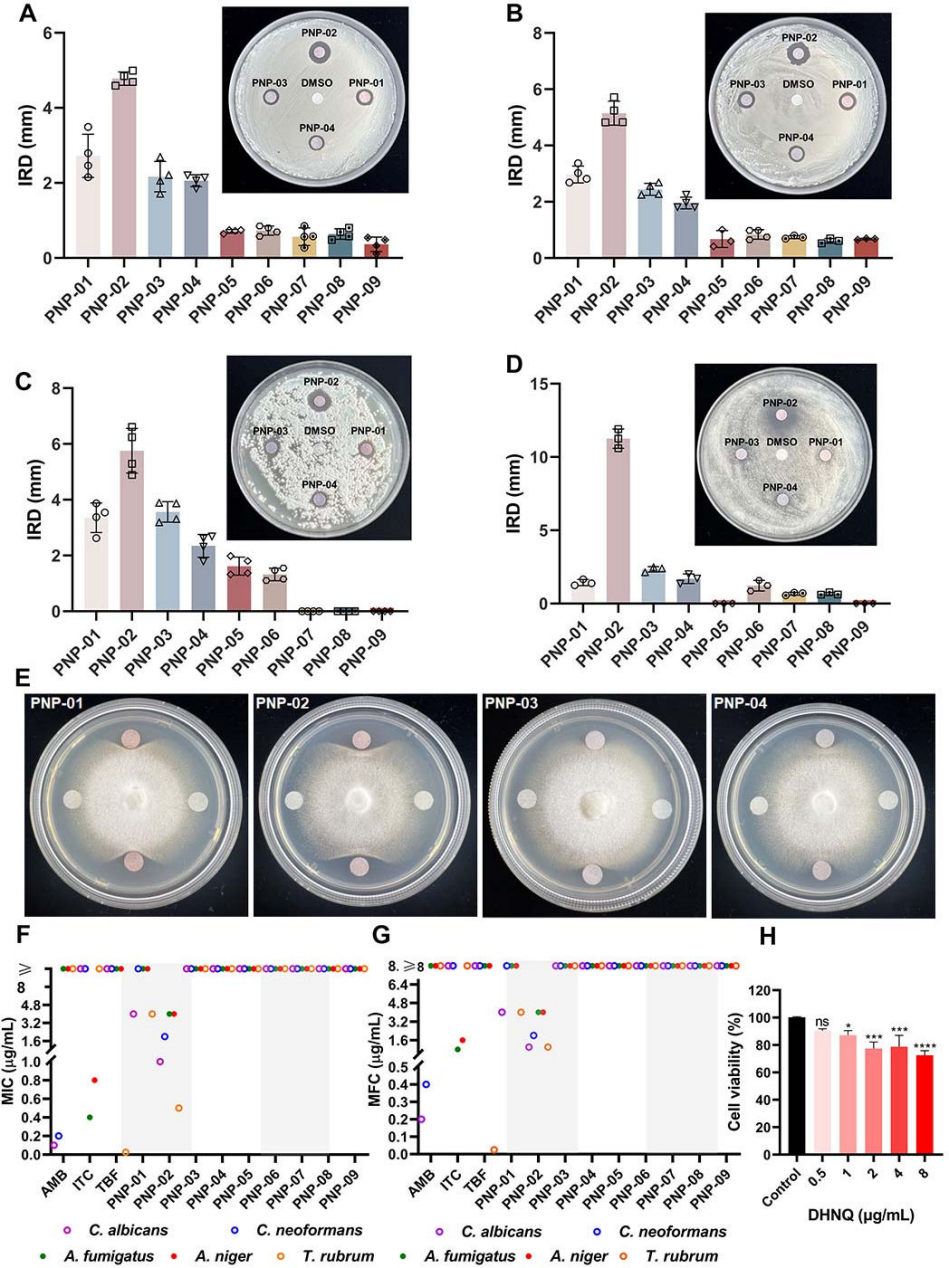
Antifungal activity of natural shikonin compounds and their parent nucleus. Antifungal activities of the compounds against (**A**) *C. albicans*, (**B**) *C. neoformans*, (**C**) *A. fumigatus*, and (**D**) *A. niger*, measured by inhibitory ring diameter (IRD). (**E**) Drug-sensitive test of *T. rubrum*. MIC (**F**) and MFC (**G**) of natural shikonin compounds and their parent nucleus against common pathogenic fungi (μg/mL). (**H**) Cytotoxicity of DHNQ against human normal cell line MCF-10A.

Using the Kirby-Bauer paper diffusion method, the anti-pathogenic fungal activity of the nine natural shikonin compounds (PNP-01 to PNP-09) and their parent nucleus were assessed (Fig. 1). A preliminary judgment of the antifungal activities of the compounds could be made based on the inhibitory ring diameter (IRD), as shown in Fig. 2A through E. PNP-01, PNP-02 (hereinafter referred to as DHNQ), PNP-03, and PNP-04 showed varying degrees of inhibition against several common pathogenic fungi, including *C. albicans*, *C. neoformans*, *A. fumigatus*, A. *niger*, and *T. rubrum* with a clear inhibitory ring.

Fig. 2F and G show that PNP-01, DHNQ, PNP-03, and PNP-04 exhibited strong antifungal activities (MIC ≤ 64 µg/mL) against five common yeasts and filamentous pathogenic fungi. Notably, for *T. rubrum*, all compounds involved in the test showed vigorous antifungal activities (MIC ≤ 32 µg/mL), which provides a theoretical basis for the feasibility of using comfrey extract as the main ingredient for treating dermatophytoses on the market. Among all tested compounds, DHNQ performed best, with MIC and MFC as low as 1 μg/mL against *C. albicans* and 2 μg/mL against *C. neoformans*, only about ten times weaker than amphotericin B, an antifungal agent used clinically to treat severe deep fungal infections. DHNQ was also a potent anti-*Aspergillus* agent, with MIC and MFC as low as 4 μg/mL against both *A. fumigatus* and *A. niger*.

### In silico analysis identified the DHNQ compound with improved antifungal activities

Several *in silico*-based prediction toolkits (OSIRIS DataWarrior, Molinspiration, and AdmetSAR) were used to predict the molecular properties, toxicity risks, bioactivities, and pharmacokinetic characteristics of PNP-01, DHNQ, PNP-03, and PNP-04. The predicted molecular properties in Table 1 indicated that all selected compounds conform to the Lipinski’s “Rule of 5” (26, 27). The toxicity risk evaluation predicted that those compounds would not be mutagenic, tumorigenic, reproductive toxic, or irritant. Moreover, we calculated the overall drug score (ranging from 0 to 1) by considering both a compound’s molecular properties and toxicity risk. Notably, PNP-01, DHNQ, PNP-03, and PNP-04 had scores higher than 0.74, outperforming several substances that tested positive.

**TABLE 1:** Calculated molecular properties, toxicity risks, and pharmacokinetic predictions of the compounds investigated^*a*^

Compounds	PNP-01	DHNQ	PNP-03	PNP-04	FCZ	AMB	ITC	TBF
Molecular properties								
MW	288.298	190.154	288.298	330.335	306.275	924.087	705.645	291.437
cLogP	2.3382	0.748	2.3382	2.8228	−0.1089	0.323	5.1512	5.3193
cLogS	−2.951	−2.25	−2.951	−3.361	−2.175	−5.077	−7.305	−6.141
nON	5	4	5	6	7	18	12	1
nOHNH	3	2	3	2	1	12	0	0
nrotb	3	0	3	5	5	3	11	4
TPSA	94.83	74.6	94.83	100.9	81.65	319.61	100.79	3.24
nviolations	0	0	0	0	0	3	3	1
Drug likeness	1.3207	0.2862	1.3207	1.6988	3.0382	−0.1375	7.616	−3.4861
Toxicity risks								
Mutagenic	None	None	None	None	None	None	High	None
Tumorigenic	None	None	None	None	None	None	High	High
Reproductive	None	None	None	None	None	None	None	None
Irritant	None	None	None	None	None	None	None	None
ADMET predictions								
HIA (probability)	0.9955	0.9951	0.9955	0.9624	0.9894	0.9308	0.9973	0.9876
CCP (probability)	0.7901	0.7166	0.7901	0.6975	0.8867	0.7539	0.5511	0.7436
CYP inhibitory promiscuity	High	Low	High	High	Low	Low	High	High
Overall drug score								
Drug score	0.7862	0.7476	0.7862	0.7581	0.9047	0.2717	0.0773	0.1311

^*a*^ MW, molecular weight; clog P: logarithm of partition coefficient between n-octanol and water; logS: aqueous solubility; nON, number of hydrogen bond acceptors; nOHNH, number of hydrogen bond donors (OH and NH groups); nrotb, number of rotatable bonds; TPSA: topological polar surface area; HIA: human intestinal absorption; CCP: Caco-2 permeability.

The natural shikonin compounds have demonstrated promising results in the fight against pathogenic fungi. The study combined antifungal activities and *in silico* calculations to identify DHNQ as the leading candidate for further research. To preliminarily assess the therapeutic potential of DHNQ, we evaluated its cytotoxicity against human normal cell line MCF-10A. As shown in Fig. 2H, DHNQ exhibited relatively low cytotoxicity toward MCF-10A at concentrations effective against *C. albicans*. As a result, DHNQ was selected for more extensive evaluations. *C. albicans*, the most common pathogenic fungus, was chosen for follow-up studies as it is more sensitive to DHNQ.

### DHNQ showed potent inhibitory activity against *C. albicans*

**Fig 3:**
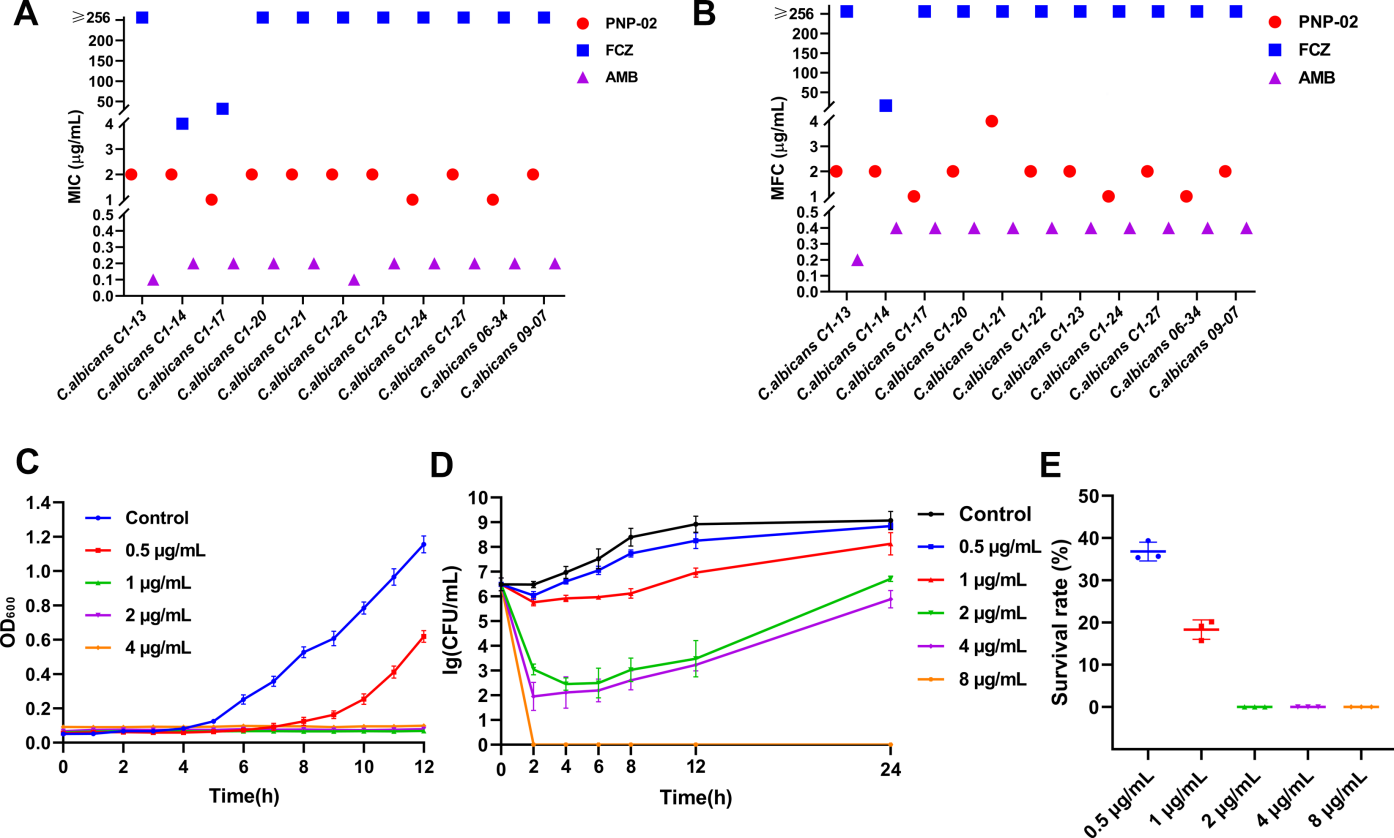
Fungicidal kinetic characteristics of DHNQ against *C. albicans*. MIC (**A**) and MFC (**B**) of DHNQ against *C. albicans* clinical isolates. (**C**) Effect of DHNQ on the growth curve of *C. albicans*. (**D**) Time-kill curve of DHNQ against *C. albicans* ATCC10231 *in vitro*. (**E**) Survival rate of *C. albicans* after 2 h of DHNQ treatment.

We tested DHNQ’s MIC and MFC against 11 clinical isolates of *C. albicans*. Figure 3A and B show that most clinical isolates tested were fluconazole-resistant strains with MIC ≥ 256 µg/mL. With an MIC of 1–2 μg/mL and an MFC of 2–4 μg/mL, DHNQ still showed strong antifungal activity against these resistant strains, which was the same as its activity against the standard *C. albicans* strain ATCC 10231.

As shown in Fig. 3C, DHNQ at 0.5 μg/mL was sufficient to affect the growth of *C. albicans*, as evidenced by a delay in the entry into the logarithmic growth phase, resulting in a significant inhibition of both growth rate and increment. The addition of DHNQ at a concentration of 1 μg/mL or higher resulted in nearly unchanged turbidity in the culture system compared to 0 h, suggesting a stronger inhibition of *C. albicans* growth.

The time-kill curves demonstrated that DHNQ had the greatest inhibitory impact within 2 h of administration, indicating that its effectiveness was time- and dose-dependent (Fig. 3D). When the concentration of DHNQ was increased to 2 μg/mL, only 0.035% of the cells survived after 2 h of treatment, and all *C. albicans* cells were killed within 2 h at a DHNQ concentration of 8 μg/mL, with no recurrence growth observed within 24 h (Fig. 3E).

### Compound DHNQ exerted potent *in vivo* antifungal activity against *C. albicans* infections

**Fig 4:**
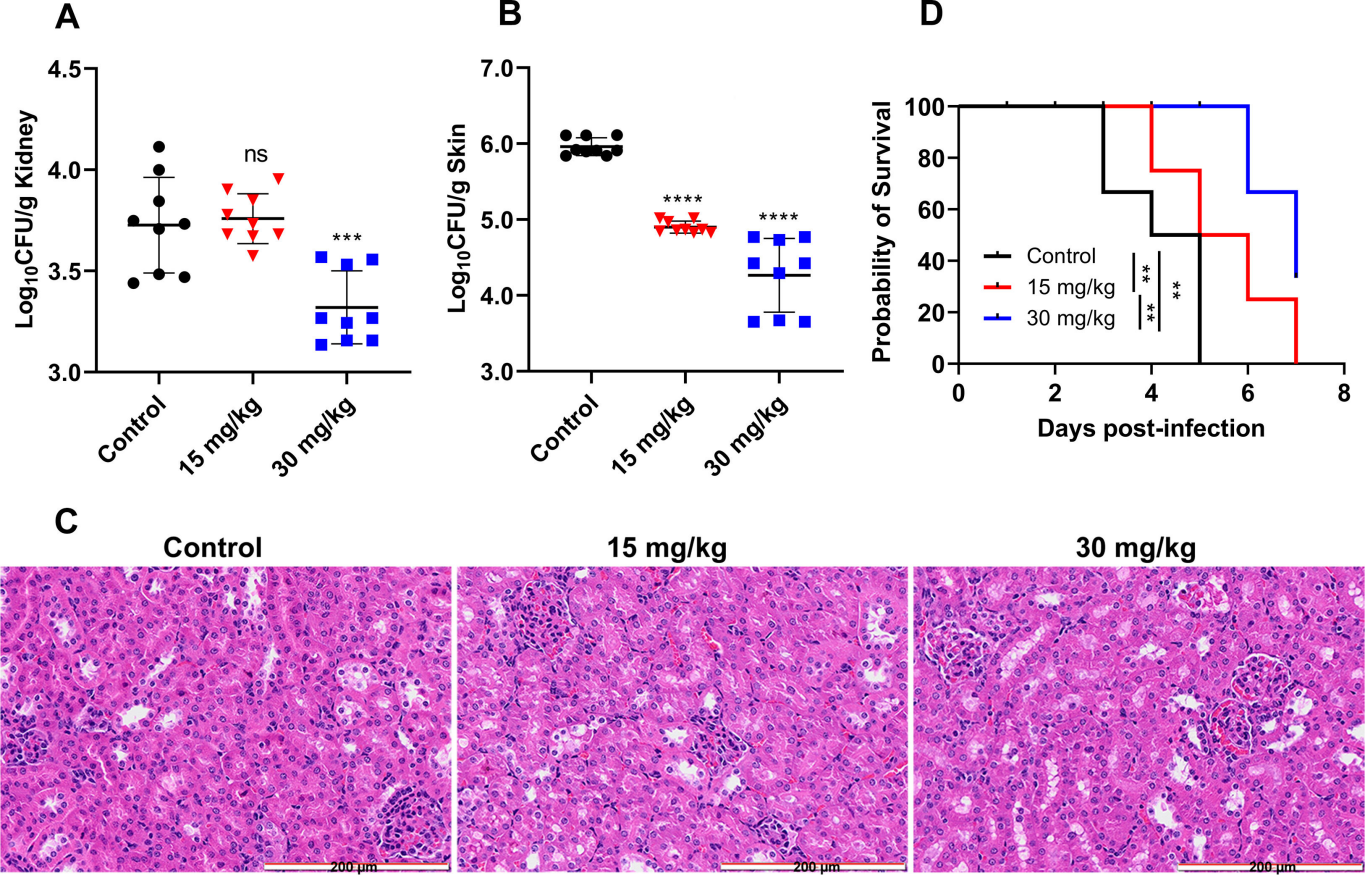
*In vivo* antifungal efficacy of compound DHNQ in deep and epidermal infections mice model. (**A**) Fungal burden of kidneys and (**B**) skin in mice infected by *C. albicans*. (**C**) Histological sections of infected mice kidney tissue with H&E staining. (**D**) Survival curves of infected mice treated with antifungal compounds. *C. albicans*-infected mice were placed into different groups (six mice per group).

The *in vitro* assays revealed that compound DHNQ exhibited excellent antifungal activities against *C. albicans* isolates. *C. albicans* mice model was established via tail vein injection of fungal cells to evaluate the *in vivo* antifungal potency of DHNQ. In the *C. albicans* infection model (Fig. 4A), oral administration of compound DHNQ at a dose of 30 mg/kg significantly reduced the fungal burden in the kidneys of mice (*P* < 0.001), which was more effective than the dose of 15 mg/kg treatment group (ns). In addition, treatment with compound DHNQ (15 mg/kg) applied to the skin wound of mice significantly alleviated the fungal burden (*P* < 0.0001), with the 30 mg/kg treatment group demonstrating improved efficacy (*P* < 0.0001) (Fig. 4B). Furthermore, histological examination of kidney tissues from treated mice revealed no signs of drug-related toxicity (Fig. 4C), supporting its potential for further therapeutic development. In the survival experiment (Fig. 4D), all mice in the control group succumbed to infection with a median survival time of 5 days. Treatment with DHNQ at 15 mg/kg and 30 mg/kg resulted in median survival times of 5.5 and 7 days, respectively. By day 7, approximately 60% of the mice in the 15 mg/kg group had died, whereas 60% of those in the 30 mg/kg group remained alive (*P* < 0.01, **). Compared to the control group, DHNQ significantly prolonged the survival of mice infected with *C. albicans* (*P* < 0.01, **). The results highlighted the potential of compound DHNQ for treating *C. albicans*.

### DHNQ destroyed the cell membrane and cell wall structure of *C. albicans*

**Fig 5:**
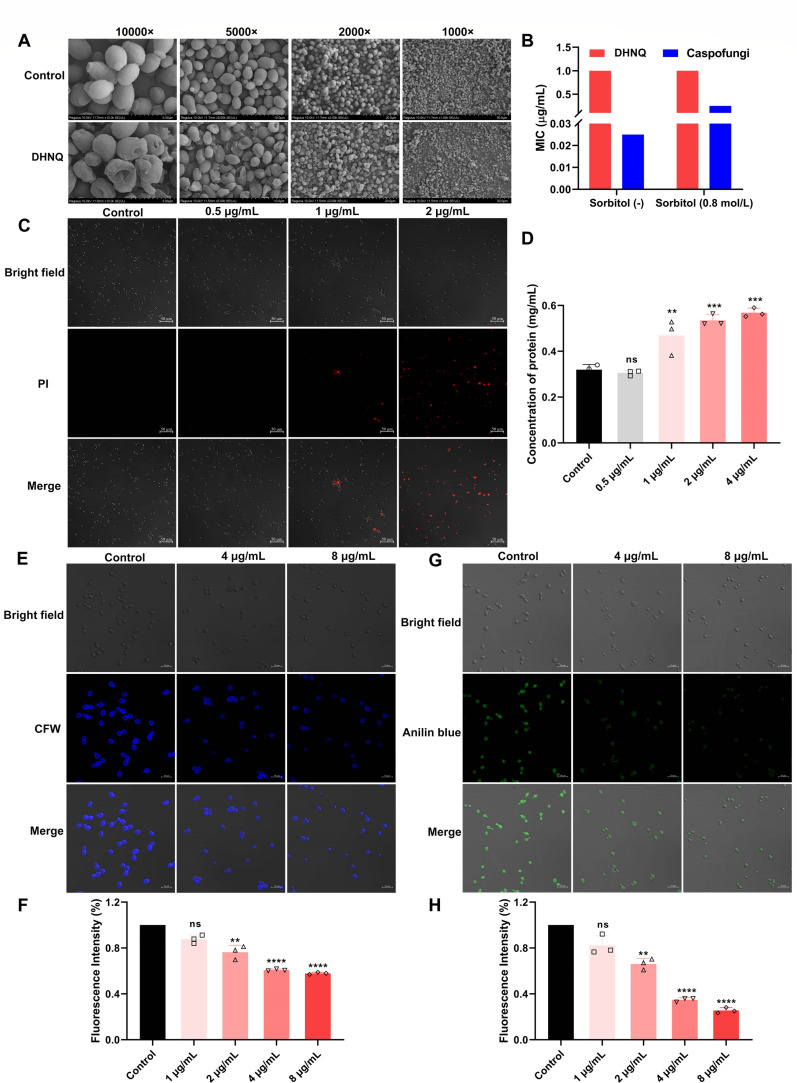
Effect of DHNQ on cell membrane integrity and cell wall of *C. albicans.* (**A**) Morphological changes of *C. albicans* treated with DHNQ. (**B**) Sorbitol protection assay. (**C**) Effect of DHNQ on membrane permeabilization of *C. albicans* (200×). (**D**) Effect of DHNQ on soluble protein of *C. albicans* medium. (**E**) Effect of DHNQ on chitin content in cell wall observed by CLSM. (**F**) Effect of DHNQ on β-1,3-glucan content in cell wall observed by CLSM. (**G**) Effect of DHNQ on chitin content in cell wall detected by fluorescent enzyme marker. (**H**) Effect of DHNQ on β-1,3-glucan content in cell wall detected by fluorescent enzyme marker. Data shown are the mean ± SD of three independent experiments (ns: no significance, **P < 0.01, ***P < 0.001, ****P < 0.0001, compared to control).

*C. albicans* cells in the control group were morphologically intact, full-bodied, and ovoid, with smooth cell surfaces and a tendency to form hyphae, as observed by confocal laser scanning microscopy (Fig. 5A). In contrast, the DHNQ-treated cells showed noticeable morphological changes. The cells displayed severe deformation, wrinkling, and distortion; a significant reduction in the budding phenomenon; and severe ruptures in some cells. DHNQ may thereby drastically alter the morphology of *C. albicans* cells, most likely because of rupturing exterior components, such as cell walls or membranes. As shown in Fig. 5C, the percentage of cells stained red by PI steadily increased with DHNQ concentration. When the concentration of DHNQ reached 2 μg/mL, almost all cells in the field of view were stained by PI, indicating that DHNQ had a vigorous activity of disrupting the cell membrane structure of *C. albicans* and affecting cell membrane permeability in a dose-dependent manner.

Large molecules, including intracellular proteins, may seep out of the cell through membrane pores when the structure of the cell membrane is severely damaged. The soluble protein concentration in the medium rose significantly in comparison to the control group following treatment with 1 μg/mL of DHNQ (Fig. 5D). When the DHNQ concentration reached 2 μg/mL and above, the level of leaking protein in the treated group increased further, again indicating that DHNQ could disrupt the integrity of the *C. albicans* cell membrane.

The chitin and β-1,3-glucan, essential cell wall components, were stained with CFW and aniline blue, respectively. Figure 5E through H shows that DHNQ could dose-dependently reduce the amount of chitin and β-1,3-glucan in the cell wall. However, as shown in Fig. 5B, unlike caspofungin, which directly targets the cell wall, the MIC of DHNQ did not rise in media containing the osmolarity stabilizer sorbitol, implying that DHNQ may not exert its antifungal action by directly targeting the synthesis of cell wall components.

### Proteomics analysis revealed that DHNQ inhibited glycolysis and oxidative stress response in *C. albicans* cells

**Fig 6:**
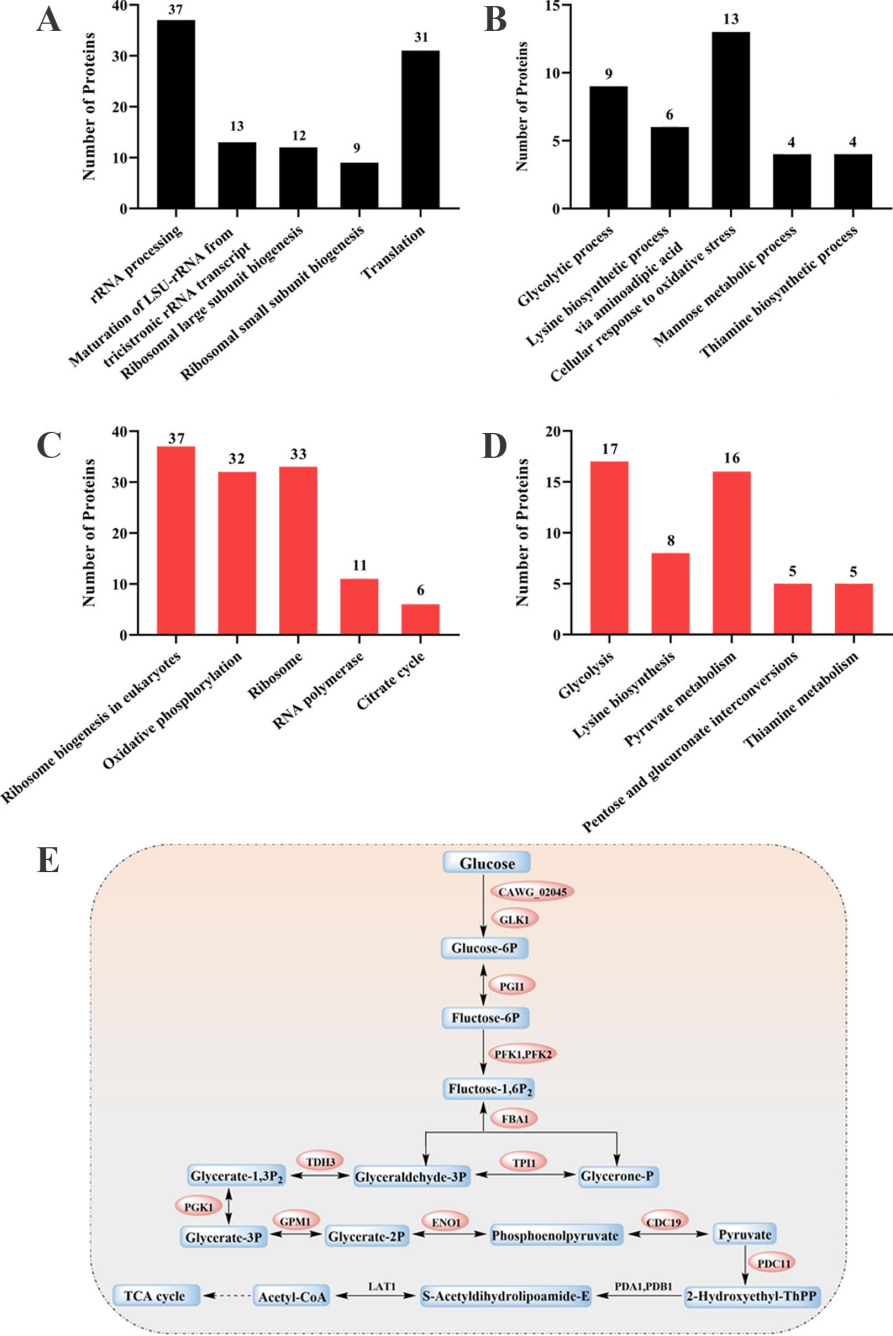
GO and KEGG analyses of differentially expressed proteins. (**A**) Top 5 GO terms of upregulated proteins. (**B**) Top 5 GO terms of downregulated proteins. (**C**) Top 5 KEGG terms of upregulated proteins. (**D**) Top 5 KEGG terms of downregulated proteins. (**E**) Glycolysis pathway after DHNQ treatment (orange: downregulated proteins; black: no differentially expressed proteins).

Further study of the antifungal mechanisms of DHNQ against *C. albicans* was conducted using proteomics to gain further insight into its global antifungal mechanisms. GO and KEGG analyses were performed on the differentially expressed proteins. Table 2 lists downregulated proteins in the oxidative stress response pathway, which are mainly involved in protecting cells from oxidative damage (see table notes for specific functions). Based on the proteomic data in Table 2, we further performed GO functional enrichment analysis. Figure 6 shows the Top 5 *P* values for each GO term and KEGG pathway, ranked from smallest to largest (i.e., significance ranked from largest to smallest). As shown in Fig. 6A and C, the significantly upregulated GO terms and KEGG pathways were associated with processes, such as rRNA processing, ribosome biogenesis, transcription, and translation, which may be related to the increased metabolic regulation and demands for essential protein synthesis caused by drug stimulation. As shown in Fig. 6B, among the GO terms significantly downregulated, the cellular response to oxidative stress had the most differentially expressed proteins. As shown in Table 2, the common function of these proteins was primarily responsible for protecting cells from oxidative damage, suggesting that DHNQ impaired the ability of *C. albicans* cells to regulate themselves in response to oxidative stress and thus being exposed to damage caused by reactive oxygen species (ROS).

**TABLE 2:** Downregulated proteins in the cellular response to oxidative stress term

Uniprot ID	Gene name	Protein name (Uniprot)	Fold change	Function	Reference
A0A1D8PF54	*PHO84*	Phosphate transporter	0.357	Associated with antioxidant and virulence	(28)
A0A1D8PHR5	*PST1*	Pst1p	0.522	Promotes the two-electron reduction reaction of quinones and prevents the production of semi-quinone radicals	(29)
A0A1D8PT02	*PST3*	Flavodoxin-like fold family protein	0.616		
A0A1D8PPI6	*CIP1*	Cip1p	0.573	Detoxification in response to cadmium stress	–^*a*^
A0A8H6BSH7	*PRX1*	Peroxiredoxin PRX1, mitochondrial	0.168	Peroxidase, involved in the balance between the reduced (GSH) and oxidized (GSSG) states of the antioxidant substance glutathione	(30)
C4YPI2	*CAWG_02383*	Thioredoxin domain-containing protein	0.471	Thioredoxin (Trx), involved in the reductive detoxification of hydrogen peroxide	(31)
Q59NQ2	*orf19.11,626*	Glutathione-disulfide reductase	0.353	Glutathione-disulfide bond reductase, which converts GSSG to GSH and provides reducing power for ROS scavenging	(32)
Q5ADM5	*orf19.14,108*	Aldo-keto reductase superfamily protein	0.537	Aldo-keto reductase, catalyzes the reduction of carbonyl groups to alcohols	–
Q5AEN1	*CCP1*	Cytochrome c peroxidase	0.515	Cytochrome C peroxidase, involved in hydrogen peroxide detoxification and methylglyoxal scavenging	(33)
Q5AFB4	*GST2*	Gst2p	0.455	Glutathione sulfotransferase, catalyzes the binding of glutathione to electrophile substances for peroxide scavenging and detoxification	(34)
Q5AH29	*orf19.13,862*	Glutaredoxin domain-containing protein	0.522	Glutoxigenin (Grx), repairs oxidative damage	(35)
Q5AHH4	*HSP21*	Small heat shock protein 21	0.522	Involved in cellular resistance to heat stress and oxidative stress, inducing hyphal growth	(36)
Q5AQ36	*SHO1*	High osmolarity signaling protein SHO1	0.598	Involved in cellular response to hyperosmotic conditions and oxidative stress	(37)

^*a*^ "–", indicates no reference cited.

The result of Fig. 6D shows that the glycolysis pathway had the highest number of differentially expressed proteins and the lowest *P* value in the downregulated KEGG pathways. As demonstrated in Fig. 6E, 11 enzymes in the glycolysis pathway were significantly downregulated after DHNQ treatment, including two critical rate-limiting enzymes, phosphotransferase (CAWG_02045, GLK1), which catalyzed the phosphorylation of glucose to glucose 6-phosphate (Glucose-6P), and pyruvate kinase (CDC19), which catalyzed the conversion of phosphoenolpyruvate to pyruvate. Thus, by inhibiting the glycolysis process and involving the tricarboxylic acid (TCA) cycle and oxidative phosphorylation from upstream, DHNQ may severely affect the utilization of glucose, an essential source of energy for *C. albicans*. This disrupts the normal physiological metabolism of the *C. albicans* cells in both material and energetic dimensions. In conclusion, in conjunction with GO and KEGG analyses, DHNQ may cause oxidative damage and limit glycolysis to achieve its antifungal effects.

### DHNQ inhibits glycolysis by reducing glucose consumption and decreasing ATP production

**Fig 7:**
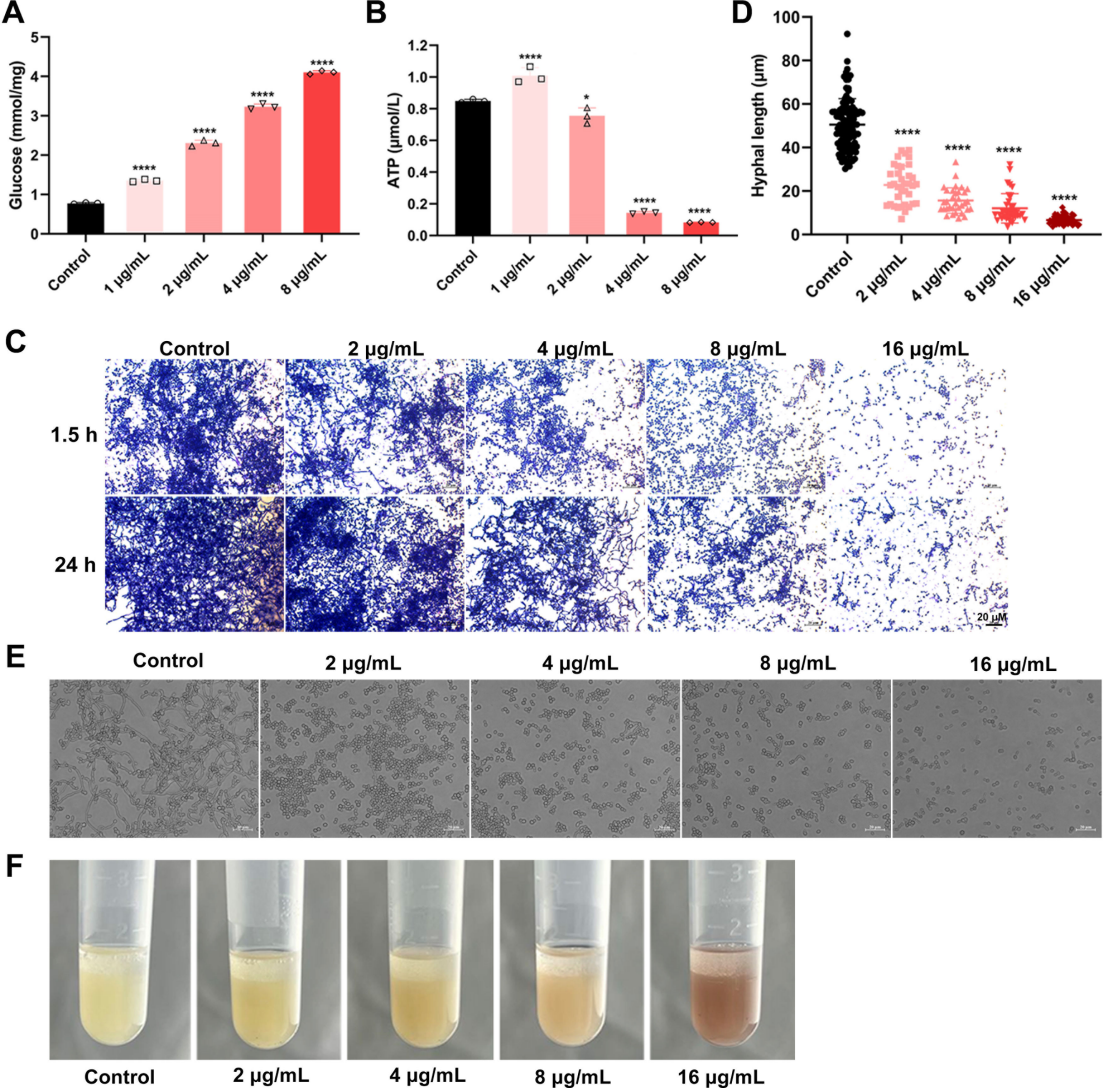
Effect of DHNQ inhibits glycolysis on *C. albicans*. (**A**) Effect of DHNQ on glucose utilization of *C. albicans*. (**B**) Effect of DHNQ on intracellular ATP of *C. albicans*. (**C**) Inhibitory effect of DHNQ on early (1.5 h) and mature (24 h) biofilm of *C. albicans*. (**D–E**) Inhibitory effect of DHNQ on hyphae growth of *C. albicans*. (**F**) Effect of DHNQ on cell surface hydrophobicity of *C. albicans* biofilm cells. Data shown are the mean ± SD of three independent experiments (ns: no significance, ****P < 0.0001, compared to control).

Although *C. albicans* has certain metabolic flexibility (38) and can use some non-glycolytic carbon sources, such as lactate, glucose is still the most crucial carbon source for its growth and metabolism, and glycolysis is the most important way to use glucose (39), which plays a key role in cellular energy and substance metabolism. The level of acetyl-CoA that enters the tricarboxylic acid cycle and oxidative phosphorylation is controlled by glycolysis, which has an impact on the synthesis of ATP in cells (40). At the same time, several glycolysis intermediate products might influence the formation of macromolecules in cells by functioning as precursors of proteins, lipids, nucleic acids, and other substances. KEGG analysis of differential proteins showed that DHNQ could downregulate multiple enzymes in the glycolytic pathway of *C. albicans*, including the vital rate-limiting enzymes phosphotransferase (hexokinase) and pyruvate kinase (40, 41). The remaining glucose concentration in the medium continued to accumulate as the DHNQ concentration increased (Fig. 7A), indicating that DHNQ inhibited the uptake of glucose by *C. albicans* cells for glycolysis and affected the use of energy substrates in a dose-dependent manner. As shown in Fig. 7B, the increased intracellular ATP levels in the 1 μg/mL DHNQ-treated group may be due to the compensatory activation of lipid catabolism and the glyoxylate cycle after glycolysis was inhibited, converting acetyl coenzyme A produced by β-oxidation of fatty acids into succinate for glucose synthesis to meet the increased energy demands under the stress of drug stimulation. However, the glyoxylate cycle’s compensatory effect was insufficient to meet cellular demands for ATP as DHNQ concentrations reached 2 μg/mL and higher, due to the increased disruption of other cellular physiological functions and suppression of glycolysis. Hence, the overall trend appears to be that DHNQ reduced cellular ATP production in a dose-dependent manner. This, together with the results of the glucose consumption assay, suggested that DHNQ severely affected the energy supply of the *C. albicans* cells by suppressing glycolysis.

### DHNQ inhibits virulence factors of *C. albicans*

Filamentation and biofilm formation have been recognized as critical virulence factors of *C. albicans*, closely related to the pathogenicity and drug resistance (42). Moreover, evidence for a strong link between glycolysis and *C. albicans* virulence has been proposed (39). To confirm whether compound DHNQ possessed anti-virulence activity against *C. albicans*, hyphae and biofilm formation assays were also performed against *C. albicans* isolates. Figure 7C shows that DHNQ disrupts *C. albicans* biofilm, inhibiting early formation and disrupting mature biofilms. In comparison, 16 μg/mL DHNQ almost completely inhibited the growth of early biofilm and disrupted 76% of the mature biofilm, indicating that DHNQ could inhibit the formation of early biofilm and disrupt the structure of mature biofilm of *C. albicans* in a dose-dependent manner.

As shown in Fig. 7D and E, cells in the control group mostly appeared as hyphal, with hyphae overlapping to form a thick network. On the other hand, the development of *C. albicans* hyphae was considerably reduced by 2 μg/mL of DHNQ. At the same time, the proportion of yeast cells in the field of view increased significantly, with fewer longer hyphae appearing at a DHNQ concentration of 16 μg/mL, and cells grew almost exclusively in the yeast state, indicating that DHNQ significantly inhibited the formation of *C. albicans* hyphae, consistent with its ability to disrupt established biofilm. The formation of Candida albicans biofilm starts with its attachment to the matrix. Cell surface hydrophobicity (CSH) contributes to the adhesion between cells and the attachment (43, 44), and CSH is positively correlated with biofilm formation (45). As shown in Fig. 7F, the n-octane phase in the control group was turbidite, indicating that there were more cells aggregated, reflecting the strong hydrophobicity of the cell surface, while the n-octane phase in the biofilm cells treated with 8 μg/mL and 16 μg/mL DHNQ was significantly clearer than that in the control group. These results indicated that DHNQ reduced the surface hydrophobicity of biofilm cells and weakened the adhesion between the cells and the support, which was also consistent with the ability of DHNQ to destroy biofilm. In addition, DHNQ demonstrates potent anti-biofilm activity against C. albicans. As shown in Table 3, the MIC and MFC of DHNQ against biofilm cells of 11 *C. albicans* clinical isolates ranged from 2 to 4 μg/mL. Compared to fluconazole, which has limited activity against the biofilm phenotype, DHNQ maintains significant antifungal efficacy against biofilm cells of fluconazole-resistant clinical isolates (MIC = 2–4 µg/mL).

**TABLE 3:** MIC and MFC of DHNQ against *C. albicans* clinical isolates biofilm cells (μg/mL)

Strains	DHNQ		FCZ		AMB	
	MIC	MFC	MIC	MFC	MIC	MFC
*C. albicans* C1-13	2	2	>256	>256	0.4	0.8
*C. albicans* C1-14	2	2	64	>256	0.2	0.4
*C. albicans* C1-17	2	2	>256	>256	0.4	0.4
*C. albicans* C1-20	2	2	>256	>256	0.4	0.4
*C. albicans* C1-21	2	2	>256	>256	0.8	0.8
*C. albicans* C1-22	2	2	>256	>256	0.2	0.4
*C. albicans* C1-23	2	2	>256	>256	0.8	0.8
*C. albicans* C1-24	2	2	>256	>256	0.4	0.8
*C. albicans* C1-27	2	2	>256	>256	0.2	0.4
*C. albicans* 06-37	2	4	>256	>256	0.4	0.4
*C. albicans* 09-07	2	2	>256	>256	0.2	0.4

### DHNQ caused oxidative stress in *C. albicans*

**Fig 8:**
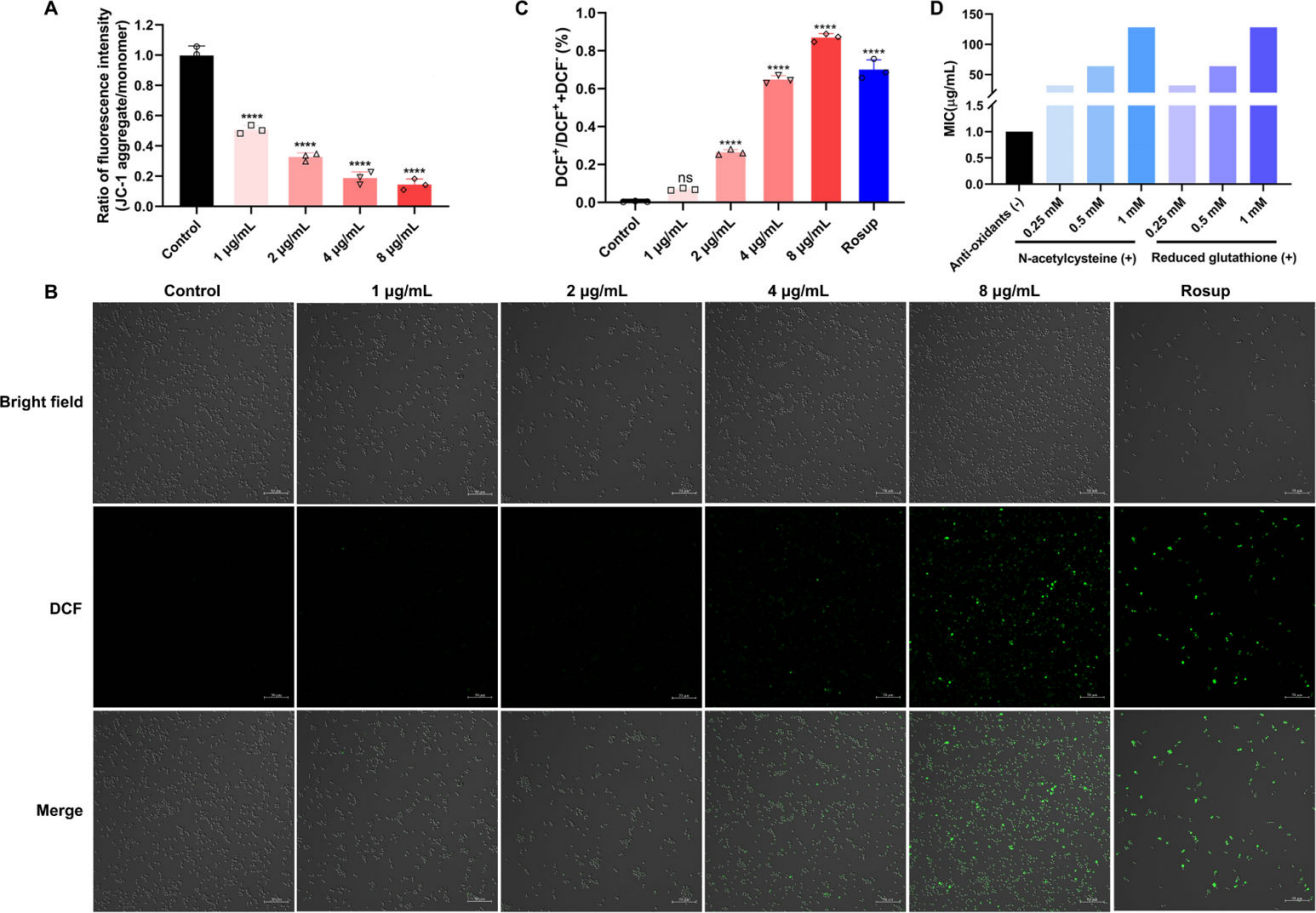
Effect of DHNQ induces oxidative stress on *C. albicans*. (**A**) Effect of DHNQ on mitochondrial membrane potential of *C. albicans.* (**B–C**) Effect of DHNQ on intracellular ROS of *C. albicans.* (**D**) MIC of DHNQ against *C. albicans* with antioxidants NAC or GSH (μg/mL). Data shown are the mean ± SD of three independent experiments (ns: no significance, ****P < 0.0001, compared to control).

The accumulation of intracellular ROS could lead to lipid peroxidation of the mitochondrial membrane (46). As shown in Fig. 8A, DHNQ significantly reduced the mitochondrial membrane potential of *C. albicans*, which was negatively correlated with the increase in ROS, further suggesting that DHNQ could exert antifungal effects by increasing intracellular ROS and causing cellular physiological damage, including mitochondrial dysfunction. As shown in Fig. 8B and C, the proportion of green, fluorescent cells in the field of view increased with DHNQ concentration. Treatment with 2 μg/mL DHNQ caused over one-fourth of the cells to be stained with DCF. In contrast, 4 μg/mL and 8 μg/mL DHNQ treatments resulted in 64.8% and 87.0% of the cells to fluoresce green, respectively, indicating that DHNQ increases intracellular ROS and causes oxidative stress in the cells.

The MIC of DHNQ against *C. albicans* was measured in a medium supplemented with different concentrations of the potent intracellular antioxidants NAC and GSH. As shown in Fig. 8D, the MIC of DHNQ against *C. albicans* was elevated 128-fold when 1 mM NAC or GSH was present in the medium. Since both NAC and GSH reacted with ROS, they reduced the level of intracellular ROS after DHNQ treatment, thereby weakening the oxidative damage caused by DHNQ.

## DISCUSSION

The increasing prevalence of *C. albicans* infections, particularly among immunocompromised patients, has highlighted the urgent need for new antifungal agents. Current antifungal therapies are limited by their spectrum of activity, side effect profiles, and the emergence of drug-resistant strains (1). This study aims to select compounds with potent antifungal activity from nine natural shikonin compounds and their parent nucleus as seedling compounds for antifungal drugs, investigate their antifungal mechanisms, provide new therapeutic options for clinical responses to fungal infections, and shed light on the research of novel antifungal drugs.

After preliminary screening and quantitative evaluation of the antifungal activities, PNP-01, DHNQ, PNP-03, and PNP-04 were found to have antifungal activity against *C. albicans*, *C. neoformans*, *A. fumigatus*, *A. niger*, and *T. rubrum* (MIC ≤ 64 µg/mL), covering the spectrum of common human pathogenic fungi, such as *Candida* species (yeast-like fungi), *Aspergillus* spp., and dermatophytes. Among the compounds tested, DHNQ demonstrated the most potent and broad-spectrum antifungal activity, with MIC less than or equal to 4 μg/mL against all five tested fungi. More notably, DHNQ maintained high antifungal activity against multiple fluconazole-resistant clinical isolates of *C. albicans* (MIC = 1–2 µg/mL), which is significantly superior to fluconazole (MIC ≥ 256 µg/mL). The results were consistent with those reported in the literature (47).

Topcu and Seker et al. demonstrated that DHNQ increases membrane permeability and disrupts membrane integrity in *C. albicans*, as evidenced by crystal violet absorption and scanning electron microscopy (47). We validated these findings using soluble protein leakage tests, PI staining, and SEM. Although *C. albicans* is a eukaryote, it possesses thick cell walls and cytoplasmic membranes that must be penetrated; this can be achieved by enhancing permeability through alterations in components such as chitin and glucan. Chitin and glucan are primarily responsible for the thick cell walls of *C. albicans* (48). Based on our findings, DHNQ considerably disrupted the normal morphological structure of *C. albicans* by affecting the synthesis of chitin and β-1,3-glucan in the cell wall, thereby impacting both the structure of the cell wall and membrane.

The antifungal mechanisms of DHNQ against *C. albicans* were further investigated through differential proteome analysis. According to GO and KEGG analyses of differentially expressed proteins, DHNQ was found to dramatically downregulate the pathways involved in glycolysis and the oxidative stress response. Although *C. albicans* has metabolic flexibility (38) and can utilize some non-glycolytic carbon sources, such as lactate, glucose remains the most critical carbon source for its growth and metabolism. Furthermore, our result indicated that DHNQ dose-dependently reduced glucose consumption in the medium by *C. albicans* cells and decreased intracellular ATP production, thereby impairing the cells’ energy supply.

Glycolysis, the central carbon metabolic pathway of the cell, is closely associated with the virulent action of *C. albicans* (39). The formation of *C. albicans* biofilm is an essential phenotype in response to their virulence, and existing antifungal drugs are largely ineffective against biofilm cells at concentrations lethal to suspension cells (49). However, DHNQ inhibited early biofilm formation and disrupted mature biofilms. Compared to the dramatic reduction in the antifungal efficacy of fluconazole on biofilm cells, DHNQ maintained similar activity to standard strains on biofilm cells of fluconazole-resistant clinical isolates. Hyphal morphogenesis is a prerequisite for biofilm to establish a complex three-dimensional structure. DHNQ also dose-dependently inhibited hyphae formation.

Resistance to oxidative stress is essential for *C. albicans* to survive properly and infect host cells. *C. albicans* has an oxidative stress response system composed of antioxidant enzymes and non-enzymatic antioxidants. It protects against oxidative stress and repairs the physiological damage caused by oxidative stress by inducing ROS detoxification enzymes and activating major antioxidant systems, including glutathione and thioredoxin systems (46). DHNQ treatment resulted in different degrees of downregulation of peroxidase, glutathione reductase, glutathione sulfotransferase, and glutathione reductase in the glutathione system, resulting in an imbalance in the ratio of reduced glutathione (GSH) to its oxidized form (GSSG), an important non-enzymatic antioxidant in the cell. In addition to inhibiting the protective mechanisms of *C. albicans* against oxidative stress, DHNQ itself contains a naphthoquinone structure that accepts electrons to form semiquinones, which are unstable and tend to transfer electrons to oxygen, thus exacerbating the production of ROS (50). Therefore, DHNQ could cause oxidative stress in *C. albicans* by simultaneously inhibiting cellular oxidative protections and inducing oxidative stress. The experimental results showed that DHNQ was able to dose-dependently exacerbate the production of ROS in *C. albicans* cells, which could lead to the dysfunction of various intracellular components, such as causing lipid peroxidation, damage to intracellular membrane structures, resulting in increased cell membrane permeability, and loss of mitochondrial membrane potential. At the same time, the antifungal effect of DHNQ was reversed by the ROS scavengers NAC and GSH.

**Fig 9:**
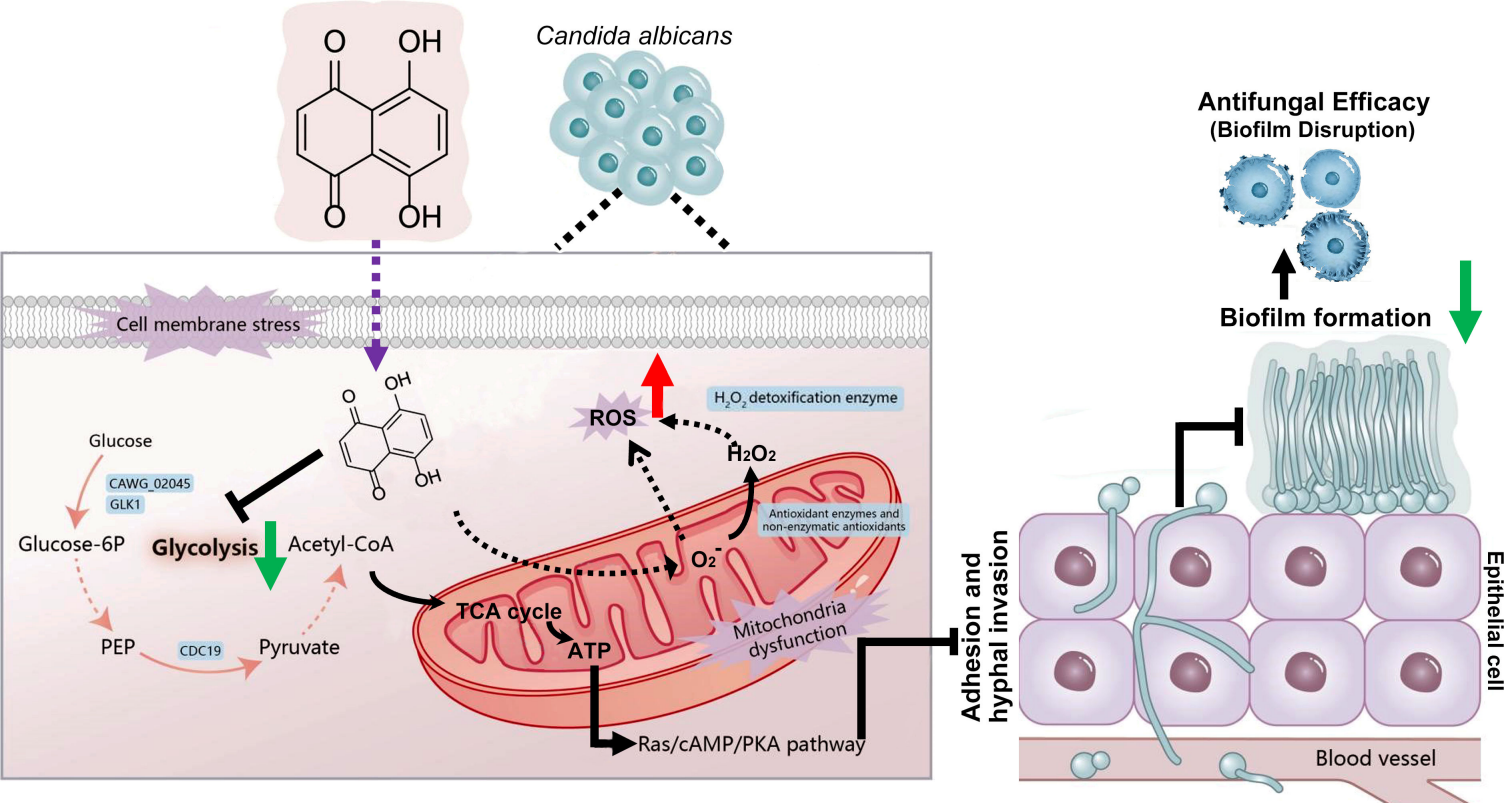
Schematic illustration of the proposed antifungal mechanism of DHNQ against *C. albicans*. Blue boxes indicate downregulated proteins identified by proteomic analysis. The diagram was created using Adobe Illustrator 2023. Red arrow: increase. Green arrow: decrease.

As summarized in our proposed model (Fig. 9), DHNQ exerts its antifungal effects through a synergistic, multi-targeted mechanism. Our integrated proteomic and functional assays demonstrate that DHNQ simultaneously attacks two fundamental pillars of cellular homeostasis. First, it inhibits key glycolytic enzymes, such as CAWG_02045 and CDC19, thereby impairing glucose consumption and depleting ATP, which disrupts cellular energy metabolism. Simultaneously, DHNQ induces severe oxidative stress by promoting ROS generation while suppressing key antioxidant defense systems, including the glutathione and thioredoxin pathways. Critically, these two pathways are not isolated but are interlinked in a vicious cycle. Mitochondrial dysfunction and membrane damage resulting from oxidative stress further compromise ATP production, while the energy crisis impairs the cell’s ability to mount an effective antioxidant response, leading to ROS accumulation. Consequently, this synergistic disruption of energy and redox balance critically compromises virulence traits, such as biofilm formation and hyphal growth. This model of a concerted attack on multiple essential cellular processes likely underlies DHNQ’s potent efficacy against drug-resistant strains and may reduce the propensity for rapid resistance development (39, 50). *In vivo*, DHNQ significantly prolonged survival in a mouse model of *C. albicans* infection model and effectively reduced fungal burden in the kidneys (deep infection) and skin (superficial infection). These findings hold considerable clinical significance, especially given the therapeutic challenges associated with deep invasive *C. albicans* infections. Furthermore, DHNQ’s activity against a critical virulence factor (biofilm), coupled with its demonstrated ability to reduce pathogen load and ultimately improve survival in an experimental model, positions it as a compelling candidate for preclinical development (2). Compared with the study by Yan et al., which used a vulvovaginal candidiasis model (14), our study employed tail vein injection-induced systemic infection models and skin incision infection models, focusing on systemic and superficial candidiasis. Despite the differences in infection sites and model construction methods employed, both studies confirm the *in vivo* anti-*Candida* activity of naphthoquinone compounds, indicating their broad potential for combatting *Candida* infections. While a relatively high concentration (8–16 μg/mL) of DHNQ was required to inhibit biofilm formation *in vitro*, the compound proved effective *in vivo*, reducing fungal load and improving the survival by inhibiting mycelia growth, inducing oxidative stress, and disrupting energy metabolism. This difference may arise from the complexity of the infection microenvironment and host immune involvement, highlighting the need for further investigation into the mechanisms underlying the observed differences in activity (51).

Although our study provides compelling evidence that DHNQ disrupts *C. albicans* by inhibiting glycolysis, inducing oxidative stress, and impairing mitochondrial function, the initial putative molecular target(s) remain to be investigated. The multifaceted phenotypic and proteomic changes we observed could stem from a single primary interaction or the simultaneous engagement of multiple targets, a common feature of many natural products. Future work employing techniques such as chemogenomic profiling in yeast deletion libraries, affinity-based pulldown assays to identify direct binding partners, or selection of DHNQ-resistant mutants for whole-genome sequencing will be crucial to pinpoint the exact protein target(s) and the upstream events that lead to the documented downstream effects. Elucidating this mechanism will advance our understanding of naphthoquinone biology and facilitate the rational development for future investigations into DHNQ’s selectivity and safety profile against host cells, which are essential to evaluate its potential as an antifungal lead compound (52).

### Conclusion

In summary, DHNQ, a compound displaying efficient and broad-spectrum antifungal activity, was screened from natural naphthoquinone compounds and showed the strongest antifungal properties against *C. albicans.* Further investigation into its antifungal mechanism was conducted through proteomic analysis and biological activity experiments, which revealed that DHNQ exerts antifungal effects by inhibiting the glycolytic pathway of *C. albicans*, disrupting biofilm structure, and suppressing hyphal growth. Additionally, it induces oxidative stress, which affects the production of intracellular ROS and results in the loss of mitochondrial membrane potential, thereby blocking the biosynthesis of virulence factors and fungal cell walls. Our findings significantly enhance the understanding of shikonin derivatives and their proposed antifungal mechanisms, providing a theoretical foundation for future clinical antifungal research.

## Data Availability

All proteomic data set obtained in this study can be accessed via Proteome X change database (https://www.iprox.cn/page/project.html?id=IPX0015450000) under accession number PXD073742.
